# Return to work after parenting in thoracic surgery: a call to action

**DOI:** 10.1093/icvts/ivae196

**Published:** 2024-11-28

**Authors:** Cecilia Pompili, Rita Costa, Isabelle Opitz, Maria Teresa Tsukazan, Niek Hugen, Nuria Novoa, Shanda Blackmon, Agathe Seguin-Givelet, Mara Antonoff

**Affiliations:** Section of Patient Centred Outcomes Research, University of Leeds, Leeds, UK; Institute for Clinical and Applied Health Research, Department of Thoracic Surgery, University of Hull, Hull, UK; Department of Cardiothoracic, Unidade Local de Saúde São João, Porto, Portugal; Department of Thoracic Surgery, Universital Hospital Zurich, Zurich, Switzerland; Department of Thoracic Surgery, Cirurgiã Torácica do Hospital Moinhos de Vento, Brazil; Thoracic Surgery Unit, Rijnstate Hospital, Arnhem, Netherlands; Department of Thoracic Surgery, University Hospital Puerta de Hierro-Majadahonda, Madrid, Spain; Thoracic Surgery Unit, Baylor College of Medicine Lung Institute Houston, Houston, Texas, USA; Department of Thoracic Surgery, Groupe Privé Hospitalier Ambroise Paré Hartmann, Paris, France; Department of Thoracic Surgery, MD Anderson Cancer Centre, Houston, TX, USA

**Keywords:** Return to work, Diversity and inclusion, Thoracic surgery

## Abstract

**OBJECTIVES:**

Women in our modern era are facing considerable challenges in the workplace, particularly in Cardiothoracic Surgery where women are under-represented in leadership and academic roles. Returning to work after parental leave may potentially uncover or exacerbate existing gender biases within the workplace, with important consequences on professional and personal lives. Our goal was to characterize the experiences and the impact of return-to-work after parenting on Thoracic Surgery careers across Europe.

**METHODS:**

All the participants of the European Society of Thoracic Surgeons Annual Meeting in 2023 were invited to complete this 32-item questionnaire. The survey was subsequently distributed through the European Society of Thoracic Surgeons social media platform from November to January 2024. A descriptive and specific group analysis was performed according to the distribution.

**RESULTS:**

There were 152 participants, 92 of whom were female (61.0%) and 66 (43%) were between 31 and 40 years, constituting the most heavily represented age group. More women parents reported their role as the primary care provider of their child (89% vs 34%; *P* < 0.00001). Moreover, presence of in-hospital childcare facilities was evaluated as more important by women. Approximately half of the parent-respondents reported breastfeeding (42, 52%), but only 26% (11) of this group indicated having any type of flexible hours for breast-feeding. Compared to men, women more often agreed that parenting might affect their career (81% vs 53%; *P* = 0.040) and felt less supported by their employers when having children (45% vs 68%; *P* = 0.37).

**CONCLUSIONS:**

This survey study identified main challenges to return to surgical work after parental leaves. Lack of structural or system-level support and limited resources for childcare and breastfeeding were considerably affecting women surgeons. Institutional initiatives for new parents and breast-feeding colleagues are crucial for supporting a diverse workforce, and any kind of discrimination derived from parental leaves should not be tolerated.

## Graphical abstract

**Figure:**
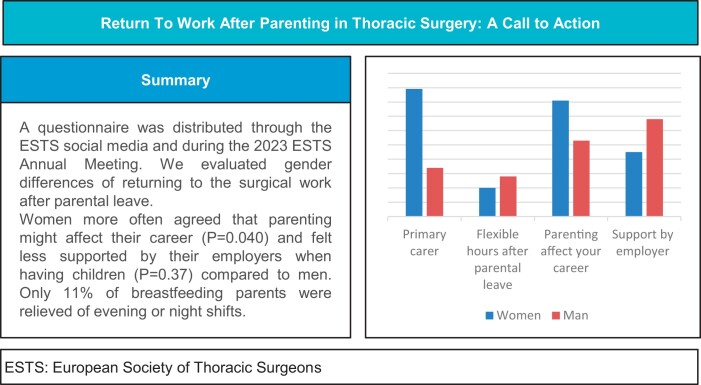

## INTRODUCTION

Women in our modern era are facing considerable challenges in the workplace that are shared by colleagues across countries and different socio-economic levels. In recent years, the role of women’s employment has been increasingly recognized as a fundamental part for a nation’s progress and expansion, impacting not just employment but also the economic, financial and social spheres [1]. The challenges and biases faced by women also extend to the professional fields like healthcare, especially those historically male-dominated, such as surgery.

In Cardiothoracic Surgery, it is broadly reported that there is a global under-representation of women and minority physicians, especially at higher leadership and academic positions. Although the proportion of graduating women medical students has increased, this rate is much slower in leadership positions, and women Full Professors will not achieve gender parity until 2136 [2].

Moreover, these concerning numbers are still present despite efforts to mitigate these issues, including mentorship programmes, leadership and personal development training [3–5]. Indeed, implicit and explicit biases are still relevant and may also be responsible for further discrimination at the financial level with a gender pay gap still particularly distressing in surgical specialities [6].

Some employers may inadvertently discriminate against parents who have stepped away to care for their children because of their perceived gaps in operative and clinical experience. This is also evident in the stigma associated with maternity leave versus military or medical leave [7]. Furthermore, individual psychological obstacles are also common, spanning from feelings of guilt related to leaving children [8] to suffering from impostor syndrome upon rejoining the professional world after time away [9].

A historically important element of family friendly policy has been the provision of maternity or parental leave rights [10]. However, returning to work after parental leave has several facets that may potentially uncover or exacerbate existing gender biases within the workplace. Surgeons who have taken time out to care for young children may face obstacles as they seek to return to operating, most notably with respect to breastfeeding [11]. The combination of returning to work and breastfeeding can be impacted by structural barriers affecting the career progression of women despite the overwhelming evidence outlining the well-established benefits of breastfeeding for both women and children, irrespective of their socioeconomic status [12]. However, employed women tend to exclusively breastfeed less than non-employed women. Indeed, returning to work is one of the major barriers to exclusive breastfeeding and breastfeeding continuation [1].

Previous work from this group has underscored and revealed a persistent underrepresentation of women in our speciality [13, 14] and has resulted in a commitment in developing and implementing initiatives and mentorship programmes to promote a diverse workforce [5, 15].

As an initial step to fulfil this commitment and identify initiatives to target, Members of the European Society of Thoracic Surgeons (ESTS) Women in General Thoracic Surgery Committee (WGTS) collaborated to design and distribute a questionnaire to characterize the experiences and the impact of return-to-work after parenting on Thoracic Surgery careers across Europe.

## METHODS

### Survey design

A survey (Supplementary Material, file 1) was developed by a subgroup of the ESTS WGTS with the support of the ESTS Student, Trainees and Early Career Members Working group (STEM).

Along with demographic information, the survey was designed to gather experiences surrounding 2 main themes: return-to-work barriers and possible facilitators that may improve work experiences after time away due to parenting. Emphasis was placed on issues relating to childcare availability and breastfeeding needs. The downstream impact of these issues on respondents’ surgical and professional career development was further explored through more additional survey items.

### Survey conduct

All the participants to the ESTS Annual Meeting in Milan on 4–6 June 2023 were invited to complete the questionnaire in person through a mobile application available at the Conference Centre after expressing their consent. The questionnaire was also distributed through the ESTS social media platform from 4 November 2023 to 1 January 2024. The questionnaire responses were collected through a link to the commercially available format (www.SurveyMonkey.com, San Mateo, CA).

### Statistical analysis

Categorical data were expressed as counts and percentages and chi-squared testing was used to analyse differences between groups.

Statistical analyses were performed using Stata (Stata Corp, College Station, TX) with significance at an alpha level of 0.05.

## RESULTS

### Participant demographics

The characteristics of the respondents are detailed in Table 1. Of 152 total survey responders, 92 (61%) were women and 66 (43%) were between 31 and 40 years, constituting the most heavily represented age group. The majority (89%) of responders work primarily in Europe with lesser representation from America (8%), Asia (1%) and Africa (1%). Thirteen of the participants in the survey did not identify the country where they work.

**Table 1:** Demographics of respondents

	Total, *n* (%)	Women, *n* (%)	Men, *n* (%)	*P*-value
Gender	152/152			
Female	92 (61)			
Male	58 (38)			
Non-binary	1 (1)			
Prefer not to say	1 (1)			
Age (years)	152/152	92/92	58/58	
20–30	31 (20)	21 (23)	10 (17)	0.005
31–40	66 (44)	46 (50)	20 (34)	
40–50	31 (20)	18 (20)	11 (19)	
>50	24 (16)	7 (8)	17 (29)	
Years post training	152/152	92/92	58/58	
On training	40 (26)	27 (29)	13 (22)	0.058
1–5	41 (27)	28 (30)	13 (22)	
6–10	27 (18)	18 (20)	8 (14)	
>10	44 (29)	19 (21)	24 (41)	
Children	152/152	92/92	58/58	
Yes	86 (57)	44 (48)	40 (69)	0.011
No	66 (43)	48 (52)	18 (31)	
Children age (years)	83/86	42/44	39/40	
0–1	24 (29)	17 (40)	7 (18)	0.060
2–5	24 (29)	14 (33)	10 (26)	
6–10	12 (14)	3 (7)	8 (20)	
10–16	10 (12)	4 (10)	5 (13)	
>16	13 (16)	4 (10)	9 (23)	
Primary carer	84/86	44/44	38/40	
Yes	53 (63)	39 (89)	13 (34)	<0.00001
No	31 (37)	5 (11)	25 (66)	

Most of the participants (86, 57%) indicated having children (Table 2), among whom more than half were younger than 5 years old (58%). The majority (53, 63%) of the participants were the primary caregiver for their child(ren).

**Table 2:** Return to work questions

	Total, *n* (%)	Women, *n* (%)	Men, *n* (%)	*P*-value
Are you or did you experience breastfeeding?	81/86	42/44	37/40	
Yes	42 (52)	38 (90)	3 (8)	<0.00001
No	39 (48)	4 (10)	34 (92)	
Do you have flexible hour arrangement at work for breastfeeding?	42/42	38/38	3/3	
Yes	11 (26)	10 (26)	1 (33)	0.792
No	31 (74)	28 (74)	2 (66)	
Is there any pumping space at work?	138/152			
Yes	22 (16)			
No	116 (84)			
Are you offered time to do breast pumping?	38/39			
Yes	10 (26)			
No	28 (74)			
Are you relieved of evenings and night shifts (calls) when breastfeeding?	41/42	37/38	3/3	
Yes	11 (26)	9 (24)	2 (66)	0.114
No	30 (74)	28 (76)	1 (33)	
Did you experience a change in milk supply when returning at work?	38/39			
Yes	28 (74)			
No	10 (26)			
Would you consider breast pumping in hospital in case of dedicated spaces?	91/94			
Yes	57 (63)			
No	24 (26)			
Do you have flexible hours at work after parental leave?	86/86	44/44	40/40	
Yes	20 (23)	9 (20)	11 (28)	0.449
No	66 (77)	35 (80)	29 (73)	
Do you think parenting might affect your career?	145/152	88/92	55/58	
Yes	101 (70)	71 (81)	29 (53)	0.040
No	44 (30)	17 (19)	26 (47)	
Do you feel supported by your employer when having children?	84/86	44/44	38/40	
Yes	46 (55)	20 (45)	26 (68)	0.037
No	38 (45)	24 (55)	12 (32)	
Did you reduce working hours due to lack of childcare at work?	83/86	43/44	38/40	
Yes	31 (37)	17 (40)	14 (37)	0.804
No	52 (63)	26 (60)	24 (63)	
Did you have to leave your job/have to decline a new work opportunity due to lack of childcare?	83/86	43/44	38/40	
Yes	25 (30)	15 (35)	10 (26)	0.405
No	58 (70)	28 (65)	28 (74)	

### Breastfeeding experiences

Approximately half of the parent-respondents reported breastfeeding (42, 52%), but only 26% (11) of this group indicated having any type of flexible hours for breastfeeding. A minority of participants in the survey had a pumping space at work (22, 16%), and 9 women and 1 non-binary person were offered time for breast pumping.

Twenty-six of the breastfeeding parents (11%) were relieved of evening or night shifts when breastfeeding. However, most of them (28, 74%) encountered changes in milk supply when returning to work. Notably, 63% (57) of the breast-pumping participants would have considered breast pumping in the hospital if they had access to dedicated spaces. When asked about their perceived importance of having a dedicated lactation space with appropriate supplies, 70% (96) answered ‘somewhat or very important’.

### Childcare facilities at work

Less than a quarter (20, 23%) of the parents reported having had flexible hours at work when they came back to clinical care after parental leave. Most of the participants (101, 70%) agreed that parenting had potentially affected their careers, although 55% (46) of the parents felt supported by their employee regarding having children.

Thirty-seven percent (31) of the parents needed to reduce working hours due lack of childcare availability at the workplace, and 30% (25) indicated that they had declined new job-related opportunities due to lack of nearby childcare facilities.

### Integration of career and parenting

Questions about the extent of agreement/relative importance were scored on a five-point Likert scale.

**Figure 1:**
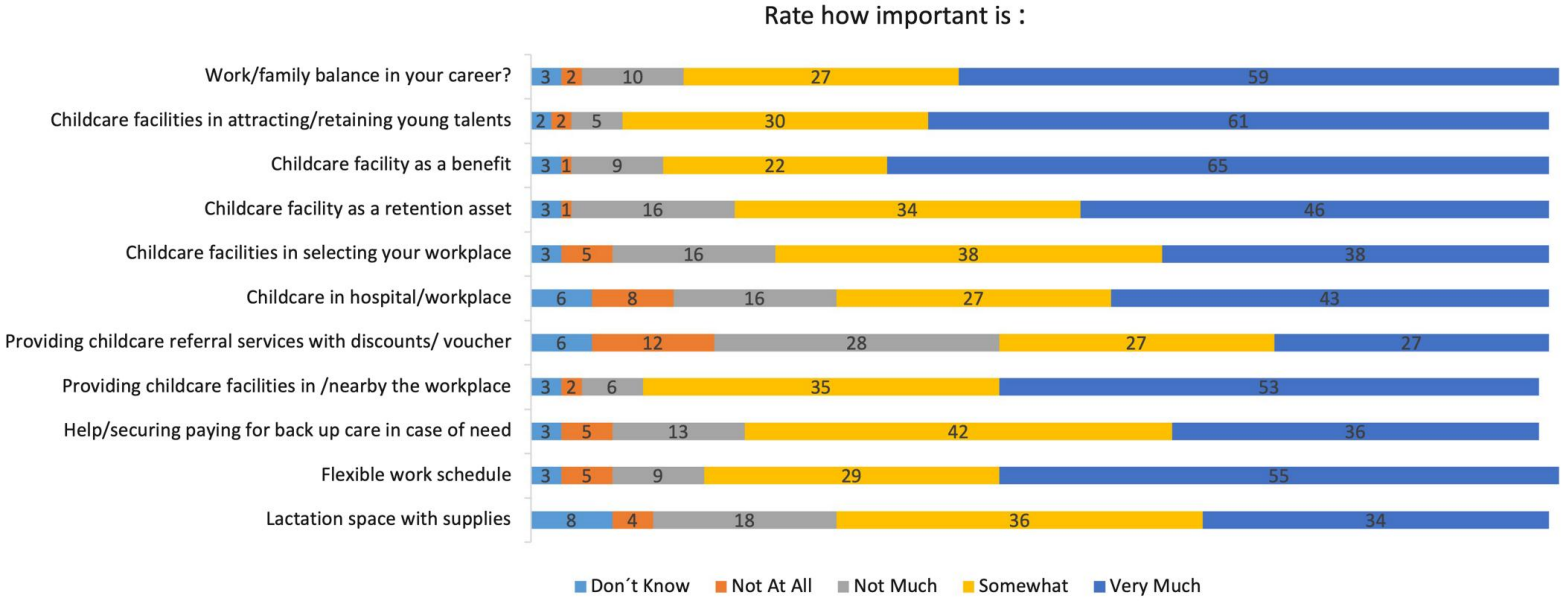
Impact of parenting on the career (all participants).

More than half (89, 59%) of the participants rated work/family balance as ‘very important’ (5/5) and 27% (41) as ‘somewhat important’ (4/5). Concordantly, 61% (93) of responders highly (5/5) recommended childcare facilities as an important element in attracting or retaining young talent, and 65% (98) rated childcare facilities as a ‘very important’ benefit (Fig. 1).

Childcare facilities were evaluated as ‘very relevant’ (5/5) assets to retain workforce by 46% (70) of the participants and as ‘somewhat important’ (4/5) by 34% (51). The importance of providing childcare nearby or in the workplace was rated for 53% (80) as ‘very important’ (5/5) and 35% (53) as ‘somewhat important’ (4/5). Flexible working hours were considered ‘very important’ (5/5) for 55% (82) of the participants and ‘somewhat important’ (4/5) for 29 (43%).

### Experiences as stratified by respondent gender

Women survey respondents were more likely to be younger than men (*P* = 0.005), with a non-significant trend towards having completed training more recently (*P* = 0.058) (Table 3). More of the men respondents were parents compared to women respondents (69% vs 48%; *P* = 0.011), yet most of the women parents noted their role as the primary care provider of their child (89% vs 34%; *P* < 0.00001).

**Table 3:** Univariate analysis comparing female and male respondents to the impact of parenting on their career questions.

	*P-value*
Work/family balance in your career?	0.385
Childcare facilities in attracting/retaining young talents	0.506
Childcare facility as a benefit	0.162
Childcare facility as a retention asset	0.784
Childcare facilities in selecting your workplace	0.030
Childcare in hospital/workplace	0.022
Providing childcare referral services with discounts/voucher	0.963
Providing childcare facilities in/nearby the workplace	0.036
Help/securing paying for back up care in case of need	0.199
Flexible work schedule	0.392
Lactation space with supplies	0.117

Furthermore, childcare facilities in selecting the workplace, childcare in workplace and providing childcare facilities in or nearby the workplace were evaluated as more important by women compared to men participants (*P* = 0.030; *P* = 0.022; *P* = 0.036, respectively).

**Figure 2:**
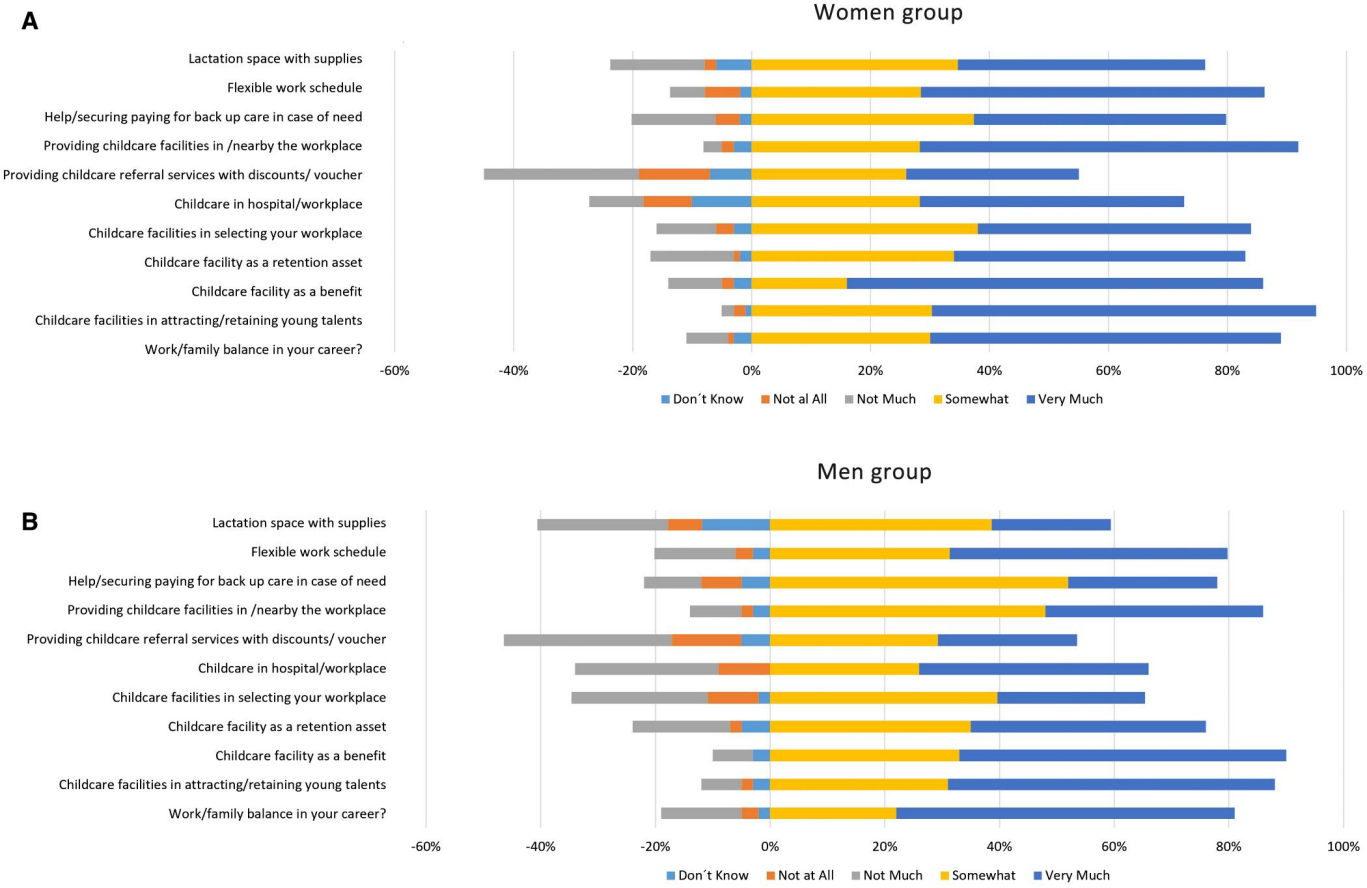
Impact of parenting on the career split by gender. (A) Women respondents. (B) Men respondents.

Compared to men, women more often agreed that parenting might affect their career (81% vs 53%; *P* = 0.040) and similarly, women felt less supported by their employers when having children (45% vs 68%; *P* = 0.037) when compared to men (Fig. 2a and b).

Nevertheless, there were no gender-related differences when only a minority of respondents reported to have taken advantage of flexible hours after parental leave, reducing working hours. There was a similar trend between the 2 genders in responding about the circumstances of declining a new work opportunity due to the lack of childcare.

## DISCUSSION

This survey clearly reported a lack of comprehensive support for new families returning to the surgical work after parental leave. The availability of in-hospital childcare facilities has been reported to have had a considerable impact on the selection of their place to work, along with breastfeeding opportunity and flexible working hours.

These 3 aspects should be the pivots for local and institutional roadmap for gender parity in surgical workplaces.

Recent reports have shown a concerning effect of significant implicit biases on clinical quality of care in the healthcare sector [16]. In this connection, the lack of female representation in our speciality has facilitated the exacerbation of implicit and explicit gender biases at multiple levels with negative effects on professional career and personal life [13, 17].

Return to work difficulties can be seen as an alarming aspect of the structural deficiencies concerning the promotion of work–life balance, especially for women. Physician mothers face a higher risk of not achieving breastfeeding goals with the main reasons identified in lack of time and appropriate place to pump breast milk, unpredictable schedule, short maternity leave and long working hours [18].

Unconscious gender bias prevents qualified women from advancing in positions of leadership and limits diversity from enhancing the quality of local and international organizations. Return to work difficulties are closely connected with lack of access to opportunities and promotions, inflexible working arrangements and employee engagement biases. Historical male-dominated surgical environments may have emphasized the lower awareness of parental entitlements and rights. The role of individual Institutions and team members can play a crucial influence in changing the biased culture in surgery [19]. Promoting inclusivity training, raising awareness of parental policies and championing breastfeeding Institutional support may facilitate progresses in championing a diverse thoracic workforce.

Considerations of parenthood can play an important role in whether individuals pursue a career in surgery, yet few reports have been recently published focusing on issues and challenges for people with parenting responsibilities working in surgery. In a recent study published in Lancet, the authors found 6 new contributing factors emerged: inaccessibility of leave, a distinction between valid and invalid reasons for leave, poor mental health, absence of interactions with other women in the surgery section, fear of repercussion and insufficient pathways for independent and specific support. Interestingly, in Australia, the number of doctors leaving surgical training considerably raised up to 2014, with significantly over-representation of women, leading to the development of the Action Plan on Discrimination, Bullying and Sexual Harassment in the Practice of Surgery [20].

ESTS WGTS has started to support the community, introducing in 2023 the first free childcare facility during the Annual Meeting. Providing easily accessible lactation rooms and childcare facilities in-hospital or close to it, while creating a culture of unbiased support, is essential for future generations of thoracic surgeons coming back to work after parenting and interested in being involved in the research.

In England, a recent House of Commons Health and Social Care Committee [21] report suggested that the parental and caring responsibilities warranted particular attention, noting that the provision of affordable and flexible childcare, flexible working and the option of a less-than-full-time working pattern are necessary for supporting participation, progression and experience in surgical careers.

Following the Kennedy report, a commissioned report has been recently released to explore the impact of parental and caring responsibilities on participation, progression and experience in surgical careers [22]. Forty percent in the Nuffield survey suggested that their parenting plans, decisions and experiences had made them less likely to pursue surgery. Similar to our results, the impact is often reported higher for women.

A range of contributory factors explain why individuals’ parenting plans, decisions and experiences influence their decision to pursue a career in surgery or their choice of specialty. However, some specialties appear to have particularly distinct challenges around the inclusion of those with parental and caring responsibilities. In part, this is due to the nature of the work as currently organized, with varying degrees of emergency or on-call work.

In our survey, male respondents were more likely to have children then women, confirming the results from a study from Ireland reporting that female trainee surgeons were almost half as likely as their male counterparts to have children [23]. Another important concern identified in this survey was a lack of support for breastfeeding (such as having places to express and store milk). Only 16% of respondents have a breastfeeding facility at their place of work. Unfortunately, a mirrored situation is found in a recently published report among surgical residents in the USA, where 67% of the 246 survey respondents stated that they did not have adequate time for pumping and 56% rarely had access to a lactation room [11].

Despite this gradual ratio increase of women in surgical residency, unique challenges facing female surgical residents who wish to start families during their training remain pronounced. The take-up of less-than-full-time training has plateaued in surgery. Outside surgery, this proportion has increased in the past 7 years up to 27%, whereas it remains at 7% in surgery [24] and most worryingly, 53.8% of those in less-than-full-time training surgical posts reported undermining behaviour as a perceived direct result of their working arrangements. However, systems must adapt to increase access to less-than-full-time training to promote trainee well-being and retention and avoid any sort of stigma or discriminatory attitudes [25].

In a culture where maximum efforts are being introduced to reduce harassment and discriminations towards women in surgery, our survey still reports lack of colleague support, referring to general comments or expressed annoyance due to a surgeon’s need to pump at work or taking less than full time working patterns [26, 27]. These attitudes have also been recently further explored in a US survey among general surgeons. This report showed that female residents were more likely to experience pregnancy/parenthood-based mistreatment with increased burnout, obstetric complications and postpartum depression [28].

### Limitations

Our study has some limitations that may have affected the results. Participation was voluntary, so there is a risk of self-selection among surgeons who chose to respond to the survey. That could explain why 69% of men and 48% of women surgeons were parents in our survey, and 61% of the respondents were female.

The survey used was not a validated questionnaire, which can have contributed to some biases. However, several rounds of focus groups within the diverse and international ESTS memberships have been performed to make sure the final instrument was iteratively refined.

Besides, our survey was distributed over social media and therefore was limited to surgeons who are active on social media and may not have reached all parent surgeons who would have participated. This has also limited the opportunity to have a precise response rate. In addition, study is a pan-European study, and the impact of different healthcare system and training regulations may have impacted the results.

## CONCLUSIONS—A CALL TO ACTION

To our knowledge, this is the 1st report indicating an urgent need to improve and standardize the return to work and breastfeeding policies in the Cardiothoracic Surgical Field. We have shown that lack of in-hospital breastfeeding support may have contributed to limit breastfeeding duration or change in milk supply for surgical residents when they came back to work. The main challenges identified were a lack of structural or system-level support, resources for childcare and support from the team, and these were more frequently identified by female surgeons.

Providing easily accessible lactation rooms and childcare facilities in-hospital or close to it, while creating a culture of unbiased support is essential for future generations of thoracic surgeons coming back to work after parental leave.

## Supplementary Material

ivae196_Supplementary_Data

## Data Availability

The data underlying this article will be shared on reasonable request to the corresponding author.

## ABBREVIATIONS

ESTS: European Society of Thoracic Surgeons
STEM: Student, Trainees and Early Career Members Working group
WGTS: Women in General Thoracic Surgery Committee
