# Supplementary material for: Return to work after parenting in thoracic surgery: a call to action
Source: Interdiscip Cardiovasc Thorac Surg. 2024 Nov 28;39(6):ivae196. doi: 10.1093/icvts/ivae196 (PMC11730188; doi:10.1093/icvts/ivae196)
Supplement: ivae196_Supplementary_Data [file ivae196_supplementary_data.pdf]

1- What is your gender?

Female
Male
Non-binary
Prefer not to say

2- How old are you?

20-30
31-40
41-50
Over 50

3- Which country do you work in?

Austria
Belgium
Bulgaria
Croatia
Cyprus
Czechia
Denmark
Estonia
Finland
France
Germany
Greece
Hungary
Iceland
Ireland
Italy
Latvia
Lithuania
Luxembourg
Malta
Netherlands
Poland
Portugal
Romania
Slovakia
Slovenia
Spain
Sweden
Switzerland
Turkiye
America
Africa
Asia
Australia
Other

4- What stage of your career are you at?

On training
1-5 years post trainng
6-10 years post training
Over 10 years post training

5- Do you have children?

Yes
No

6- How old is your child? (the youngest if you have more then one)

0-1 year
2-5 years
6-10 years
11-16 years
Over 16 years

7- How important is work/family balance in your career?

1= Don't know
2= Not at all
3= Not much
4= Somewhat
5= Very much

8- Would you recommend childcare facilities as an important element in attracting/retaining young talents?

1= Don't know
2= Not at all
3= Not much
4= Somewhat
5= Very much

9- Do you consider childcare facility as a benefit?

1= Don't know
2= Not at all
3= Not much
4= Somewhat
5= Very much

10- Do you consider childcare facility as a retention asset?

1= Don't know
2= Not at all
3= Not much
4= Somewhat
5= Very much

11- How important is/would be childcare facilities in selecting your workplace?

1= Don't know
2= Not at all
3= Not much
4= Somewhat
5= Very much

12- Do you have flexible hours at work after parental leave?

Yes
No

13- Do you have flexible hour arrangement at work for breastfeeding?

Yes
No

14- Rate how important is for you childcare in hospital/workplace

1= Don't know
2= Not at all
3= Not much
4= Somewhat
5= Very much

15- Rate how important is for you providing childcare referral services with discounts/ voucher

1= Don't know
2= Not at all
3= Not much
4= Somewhat
5= Very much

16- Rate how important is for you providing childcare facilities in /nearby the workplace

1= Don't know
2= Not at all
3= Not much
4= Somewhat
5= Very much

17- Rate how important is for you help/securing paying for back up care in case of need

1= Don't know
2= Not at all
3= Not much
4= Somewhat
5= Very much

18- Rate how important is for you flexible work schedule

1= Don't know
2= Not at all
3= Not much
4= Somewhat
5= Very much

19- Are you the primary carer of your child?

Yes
No

20- Do you think parenting might affect your career?

Yes
No

21- Do you feel supported by your employee when having children?

Yes
No

22- Did you reduce working hours due to lack of childcare at work?

Yes
No

23- Did you have to leave your job / have to decline a new work opportunity due to lack of childcare?

Yes
No

24- Are you or did you experience breastfeeding?

Yes
No

25- Is there any pumping space at work?

Yes
No

26- If YES, please describe how is it equipped.

27- Are you offered time to do breastpumping?

Yes
No

28- Are you relieved of evenings and night shifts (calls) when breastfeeding?

Yes

No

29- Did you experience a change in milk supply when returning at work?

Yes

No

30- Would you consider breastpumping in hospital in case of dedicated spaces?

Yes

No

31-rate how important is for you a lactation space with supplies

1= Don't know
2= Not at all
3= Not much
4= Somewhat
5= Very much

32- Rate how important is for you a Flexible schedule

1= Don't know
2= Not at all
3= Not much
4= Somewhat
5= Very much
